# Apixaban for the treatment of cancer-associated venous thromboembolism and left atrial appendage thrombus refractory to optimal anticoagulation with warfarin: a case report

**DOI:** 10.1093/ehjcr/yty135

**Published:** 2018-11-26

**Authors:** Masashi Koga, Atsuhiko Sugimoto, Masaki Furo, Harukazu Iseki

**Affiliations:** Department of Cardiovascular Medicine, Sagamihara Kyodo Hospital, Midori-Ku, Hashimoto, Sagamihara, Kanagawa, Japan

**Keywords:** Cancer-associated venous thromboembolism, Left atrial appendage thrombus, Factor Xa inhibitors, Direct oral anticoagulants, Case report

## Abstract

**Background:**

Concomitant venous thromboembolism (VTE) and left atrial appendage (LAA) thrombus associated with cancer is exceedingly rare. The use of direct factor Xa inhibitors in patients with cancer is controversial.

**Case summary:**

We report a rare case of concomitant VTE and LAA thrombus in an 85-year-old man with prostate cancer. He developed VTE and LAA thrombus, while on warfarin therapy for non-valvular atrial fibrillation. Despite optimal medical treatment with warfarin, systemic thrombosis developed. After thrombolysis, he was prescribed apixaban, an oral direct factor Xa inhibitor, as maintenance therapy. Deep venous thrombosis, pulmonary embolism, and LAA thrombus were effectively treated, and his symptoms resolved.

**Discussion:**

Despite the fact that many patients with cancer are in a hypercoagulable state, to the best of our knowledge, this is a first case describing VTE and LAA thrombus presenting concomitantly during optimal warfarin therapy. This case demonstrates the importance of awareness of systemic thrombosis in patients with cancer regardless of vitamin K antagonist therapy. More cases and larger scale data are needed to investigate if factor Xa inhibitors are useful for treating systemic thrombosis in patients with cancer.

Learning points

- Systemic thrombosis can develop in patients with cancer despite being on optimal warfarin anticoagulation therapy.
- Direct oral anticoagulants (DOACs) have been reported to be as safe and effective as conventional therapy for treating venous thromboembolism in patients with cancer. On the other hand, the effect of DOACs on left atrial appendage thrombi is not well understood.
- More cases and larger scale data are needed to investigate if factor Xa inhibitors are useful for treating systemic thrombosis in patients with cancer.

## Introduction

Venous thromboembolism (VTE) is the third leading cause of cardiovascular mortality.^1^ Venous thromboembolism commonly occurs in patients with cancer. Left atrial appendage (LAA) thrombi are associated with non-haemorrhagic stroke and systemic embolic events in patients with non-valvular atrial fibrillation (AF). Vitamin K antagonists (VKAs) were the standard of care for patients with VTE or AF. Direct oral anticoagulants (DOACs) are approved to treat VTE and prevent ischaemic stroke in patients with AF. There are no previous reports of simultaneous VTE and LAA thrombus during optimal anticoagulation treatment with VKAs.

## Timeline

**Table:** 

Time	Event
3 years ago	Prostate cancer and atrial fibrillation were diagnosed
Over the past 2 years	The patient’s international normalized ratio on warfarin was almost always within the therapeutic range Prostate cancer was in remission with medical treatment
Day 1	The patient presented with left lower extremity oedema. Enhanced computed tomography (CT) confirmed pulmonary embolism (PE), deep vein thrombosis (DVT), and left atrial appendage (LAA) thrombus. Anticoagulation was switched to unfractionated heparin instead of warfarin
Day 10	Because of his refractory symptoms, systemic thrombolysis with urokinase was initiated
Day 17	Ultrasonography of the lower extremities showing no residual DVT The patient was prescribed apixaban
Follow-up (1 month)	Follow-up CT imaging revealed complete resolution of PE, DVT, and LAA thrombus
Outpatient clinic (12 months)	Marked improvement in the patient’s clinical status. No recurrence of venous thromboembolism and LAA thrombus

## Case presentation

An 85-year-old man was referred to our hospital for the treatment of left lower extremity oedema. His past medical history included localized prostate cancer and cardiogenic cerebral infarction caused by AF. He was taking warfarin (1.5 mg daily) at the time. His prothrombin time-international normalized ratio (PT-INR) was checked monthly by his primary care physician. It was almost always between 2 and 3 over the past 2 years. The most recent PT-INR, approximately 2 months before admission, was 2.66. Three years ago, after prostate cancer was diagnosed, an anti-androgenic agent and a luteinising hormone-releasing hormone agonist were started. Prostate cancer was in remission with these medicines. He had no other risk factors for VTE.

On admission, he was afebrile, heart rate was 96 b.p.m., blood pressure was 170/104 mmHg, and respiratory rate was 24 b.p.m. His oxygen saturation was 96% on room air. There was no obvious jugular venous distention or audible murmurs. The lung fields were clear. His left lower extremity was erythematous and swollen.

**Figure 1:**
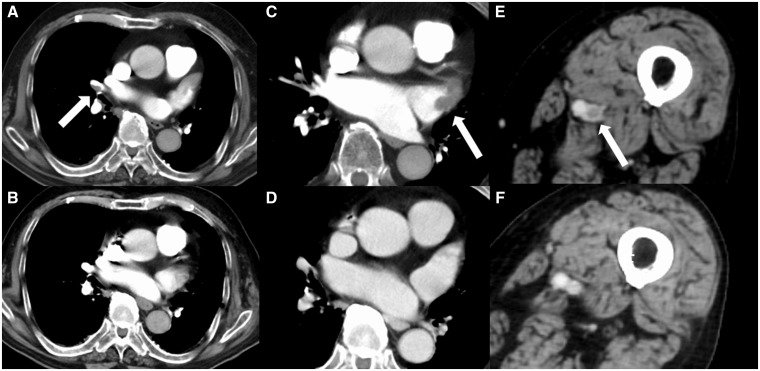
Time course of the pulmonary embolism (*A* and *B*), left atrial appendage thrombus (*C* and *D*), and deep venous thrombosis (*E* and *F*) on contrast-enhanced computed tomography. (*A*) A thrombus was detected in the right pulmonary artery (arrow). (*B*) This thrombus had completely disappeared by 1 month after discharge. (*C*) A thrombus was detected in the left atrial appendage (arrow). (*D*) No thrombi were detected at 1 month after discharge. (*E*) A thrombus was detected in the left superficial femoral vein (arrow). (*F*) No thrombi were detected at 1 month after discharge.

Laboratory tests revealed high levels of inflammation, as evidenced by the high white blood cell count, 11 100/μL (reference range 3500–8000/μL); C-reactive protein level, 7.55 mg/dL (<0.2 mg/dL); and d-dimer level, 37.0 μg/dL (<1.0 μg/dL). He had normal antinuclear antibody titres. Lupus anticoagulant, anticardiolipin IgG antibodies, and anti-β2-glycoprotein titres were negative. His serum creatinine level was 0.86 mg/dL (0.5–1.2 mg/dL) and his creatinine clearance was 62 mL/min (70–130 mL/min). At presentation, PT-INR was 3.75 (0.9–1.1). Electrocardiography showed AF and inverted T waves in V1 and V2. Computed tomography (CT) with contrast revealed intraluminal filling defects in the LAA, right pulmonary artery, and from the left superficial femoral vein (SFV) to the left popliteal vein (*Figure 1A, C, E*).

Anticoagulation was switched from warfarin to unfractionated heparin (UFH). The target activated partial thromboplastin time was 60–80 s (25–40 s). We administered UFH for 1 week, with no changes in symptoms. No obvious decrease in the size of the thrombi was observed on follow-up CT. Given the patient’s refractory symptoms, refusal to undergo catheter-directed thrombolysis, and no absolute contraindications to urokinase, we decided to perform systemic thrombolysis (360 000 to 540 000 units/day of urokinase) for 1 week to improve his acute lower extremity symptoms and prevent post-thrombotic syndrome. He was then switched to a DOAC after complete symptom resolution and ultrasonography showed no lower extremity thrombi. The direct factor Xa inhibitor apixaban was started at 5 mg twice daily. Enhanced CT approximately 1 month after hospital discharge showed complete resolution of the LAA thrombus, deep vein thrombosis (DVT), and pulmonary embolism (*Figure 1B, D, F*).

During 12 months of follow-up, the patient was doing well with significant improvement in his quality of life. Venous thromboembolism and LAA thrombus were not detected by enhanced CT at 12 months.

## Discussion

The pathogenesis of cancer-related thrombosis is poorly understood, but it is likely to be multifactorial. Virchow’s triad is thought to play a central role in the induction of thrombosis, which may be accompanied by other comorbid factors that increase hypercoagulability. Nakamura *et al*.^2^ showed that 27.0% of patients with VTE in Japan have a history of cancer. Low molecular weight heparins (LMWHs) alone have been shown to be more effective and as safe as LMWH followed by VKAs. Thus, LMWHs have become the standard of care for treating VTE in patients with cancer, but cannot be used for VTE in Japan because of insurance coverage issues. In addition, long-term injection therapy is associated with substantial costs and injection fatigue. In recent years, DOACs have been reported to be as safe and effective as conventional VTE therapy in patients with cancer. Vedovati *et al*.^3^ conducted a meta-analysis of randomised controlled trials that assessed the safety and efficacy of DOACs in patients with cancer-associated VTE. In their study, VTE recurred in 3.9% of patients with cancer treated with DOACs, compared with 6.0% patients who received conventional treatment (odds ratio 0.63; 95% confidence interval 0.37–1.10). Direct oral anticoagulants are an attractive alternative for treating VTE in patients with cancer. In this case, we selected a DOAC as maintenance therapy.

It is important to reduce thrombus volume during early stages of treatment. Thus, we performed thrombolysis prior to DOAC administration. Schweitzer *et al*.^4^ reported that systemic thrombolytic therapy results in significantly fewer closed vein segments 12 months after acute DVT than conventional treatment. They administered urokinase for up to 7 days, while monitoring for bleeding and lower extremity symptoms. Similarly, we administered urokinase for 7 days and frequently evaluated the extent of thrombus dissolution using ultrasonography. When thrombus was no longer detected in the left SFV on Day 7, thrombolysis was discontinued.

On the other hand, most thromboembolisms associated with AF originate in the LAA. The effect of DOACs on LAA thrombi is not well understood. There are several case reports about the effects of DOACs on LAA thrombi,^5–9^ but no meta-analyses or prospective studies. To the best of our knowledge, there have been no previous reports of simultaneous VTE and LAA thrombosis during optimal anticoagulation treatment with VKAs.

Warfarin and factor Xa inhibitors have different mechanisms of action. Warfarin inhibits vitamin K-dependent coagulation factors (factors II, VII, IX, and X). It also inhibits vitamin K-dependent gamma-carboxylation of proteins C and S; both have anticoagulant effects. On the other hand, factor Xa inhibitors do not consume proteins C and S. Instead, they have complementary anticoagulant actions. The mechanisms underlying hypercoagulability in patients with malignancy are not sufficiently understood. Tumour-specific activation of factor X may be an important step in the activation of the coagulation cascade in patients with malignancy.^10^ Factor Xa inhibitors might be effective for treating systemic thrombosis during VKA therapy.

We chose apixaban for the following reasons. First, apixaban has higher trough concentrations and smaller peak-to-trough fluctuations in plasma as well as anti-factor Xa activity.^11^ Second, factor Xa inhibitors reportedly do not change levels of existing thrombin and will not completely suppress thrombin production.^12^ Therefore, a small amount of thrombin might be sufficient to activate high-affinity thrombin receptors on platelets to maintain haemostasis. Miwa *et al*.^6^ reported the case of a patient in whom apixaban resolved LAA thrombus refractory to warfarin and dabigatran.

This case demonstrates the importance of awareness of systemic thrombosis in patients with cancer regardless of VKA therapy. More cases and larger scale data are needed to investigate if factor Xa inhibitors are useful for treating systemic thrombosis in patients with cancer.

**Slide sets:** A fully edited slide set detailing this case and suitable for local presentation is available online as Supplementary data.

**Consent:** The author/s confirm that written consent for submission and publication of this case report including image(s) and associated text has been obtained from the patient in line with COPE guidance.

**Conflict of interest:** none declared.

## Supplementary Material

Supplementary Data
