# Supplementary material for: Apixaban for the treatment of cancer-associated venous thromboembolism and left atrial appendage thrombus refractory to optimal anticoagulation with warfarin: a case report
Source: Eur Heart J Case Rep. 2018 Nov 26;2(4):yty135. doi: 10.1093/ehjcr/yty135 (PMC6426013; doi:10.1093/ehjcr/yty135)
Supplement: Supplementary Data [file yty135_supp.pptx]

## Slide 1
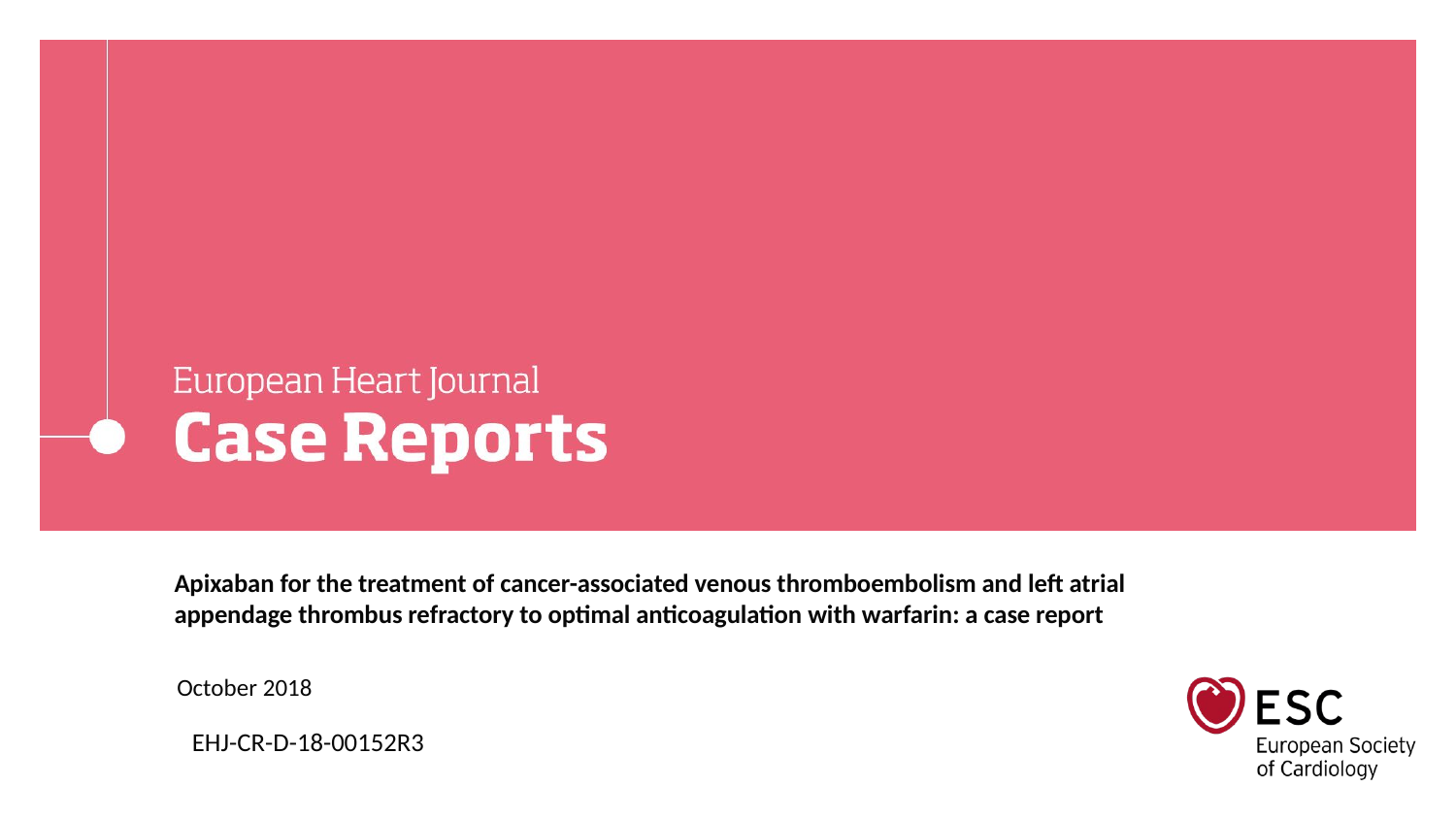

# Apixaban for the treatment of cancer-associated venous thromboembolism and left atrial appendage thrombus refractory to optimal anticoagulation with warfarin: a case report
October 2018
EHJ-CR-D-18-00152R3

## Slide 2
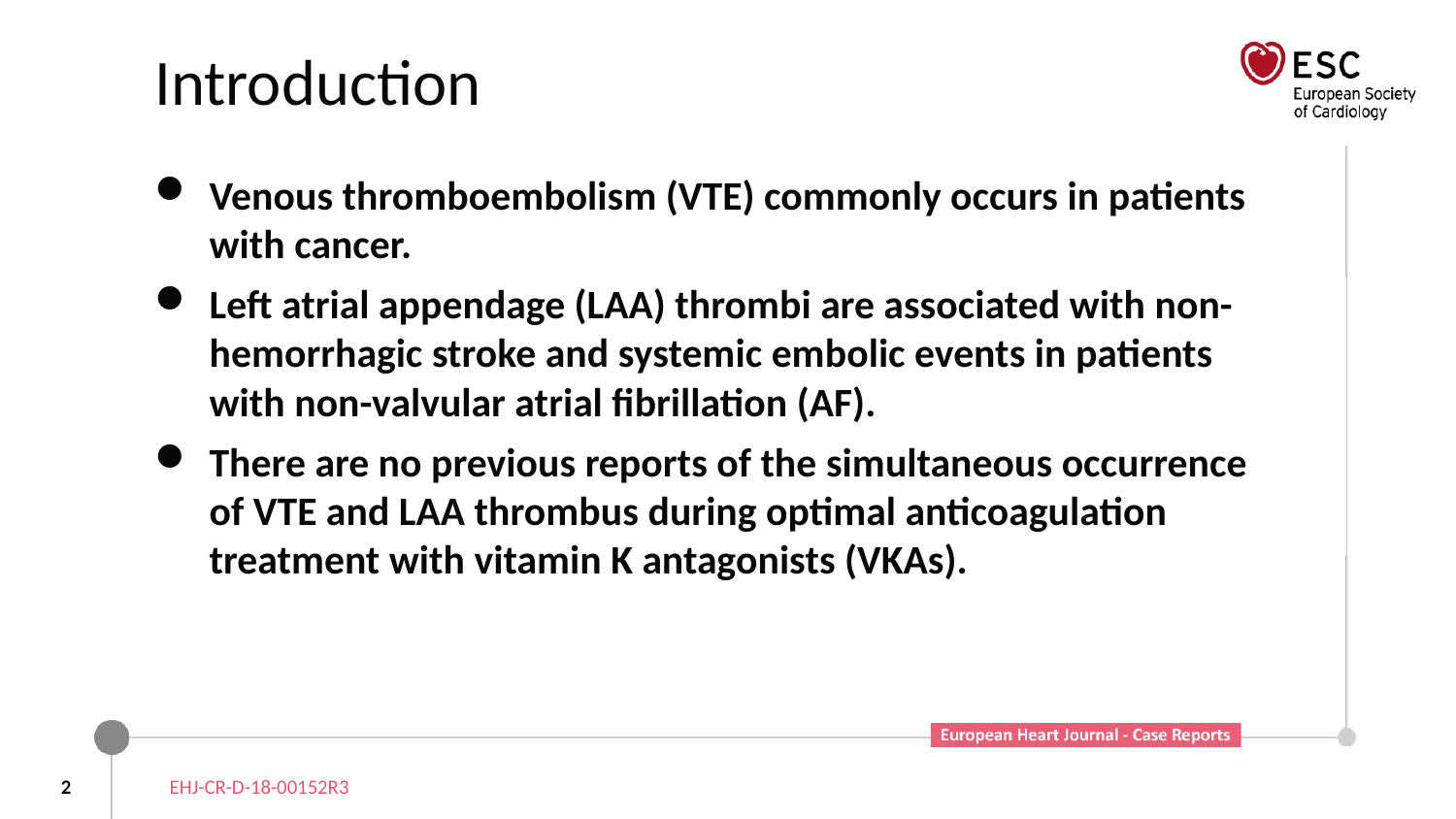

# Introduction
Venous thromboembolism (VTE) commonly occurs in patients with cancer.
Left atrial appendage (LAA) thrombi are associated with non-hemorrhagic stroke and systemic embolic events in patients with non-valvular atrial fibrillation (AF).
There are no previous reports of the simultaneous occurrence of VTE and LAA thrombus during optimal anticoagulation treatment with vitamin K antagonists (VKAs).
2
EHJ-CR-D-18-00152R3

## Slide 3
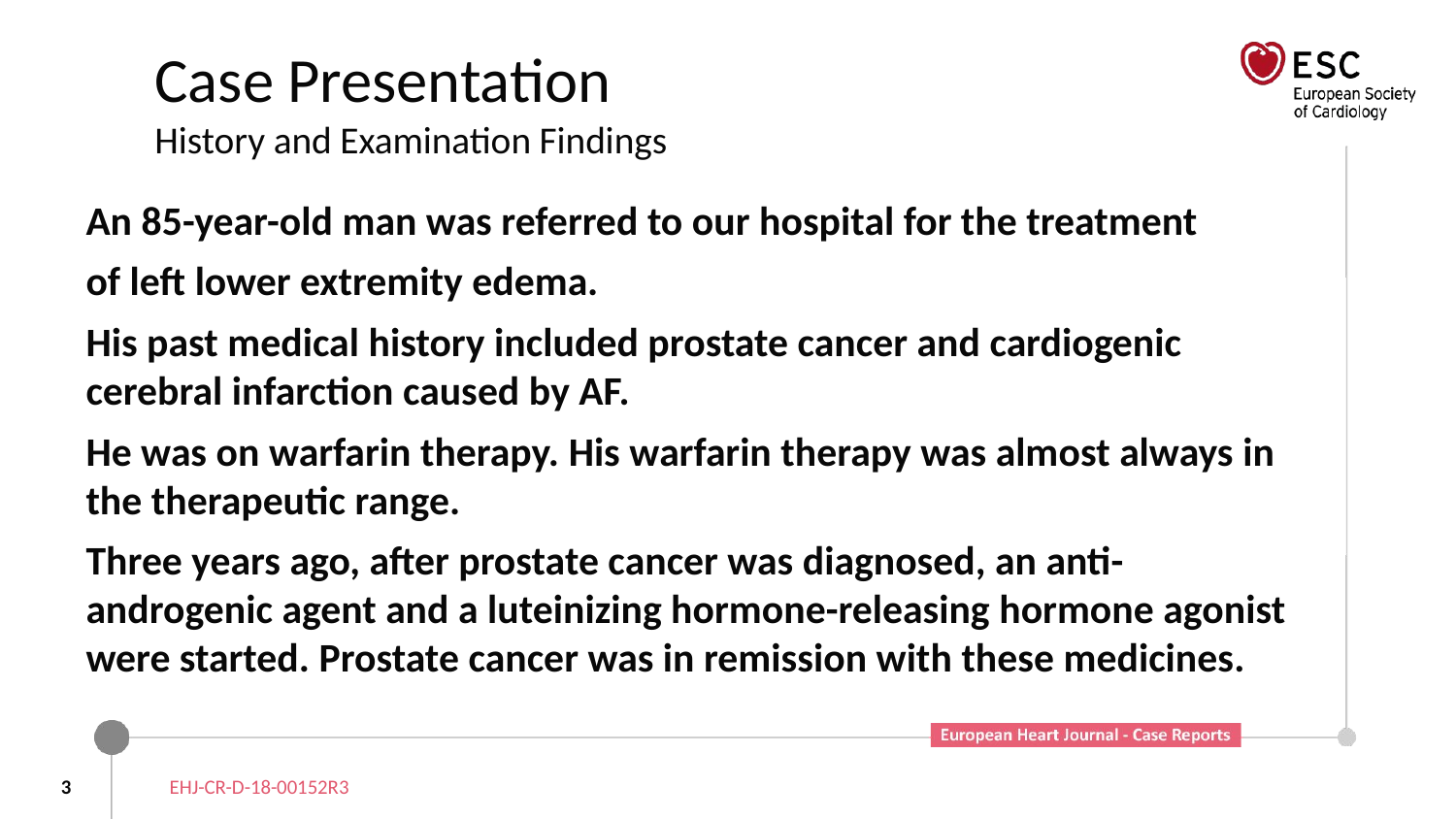

# Case Presentation History and Examination Findings
An 85-year-old man was referred to our hospital for the treatment
of left lower extremity edema.
His past medical history included prostate cancer and cardiogenic cerebral infarction caused by AF.
He was on warfarin therapy. His warfarin therapy was almost always in the therapeutic range.
Three years ago, after prostate cancer was diagnosed, an anti-androgenic agent and a luteinizing hormone-releasing hormone agonist were started. Prostate cancer was in remission with these medicines.
3
EHJ-CR-D-18-00152R3

## Slide 4
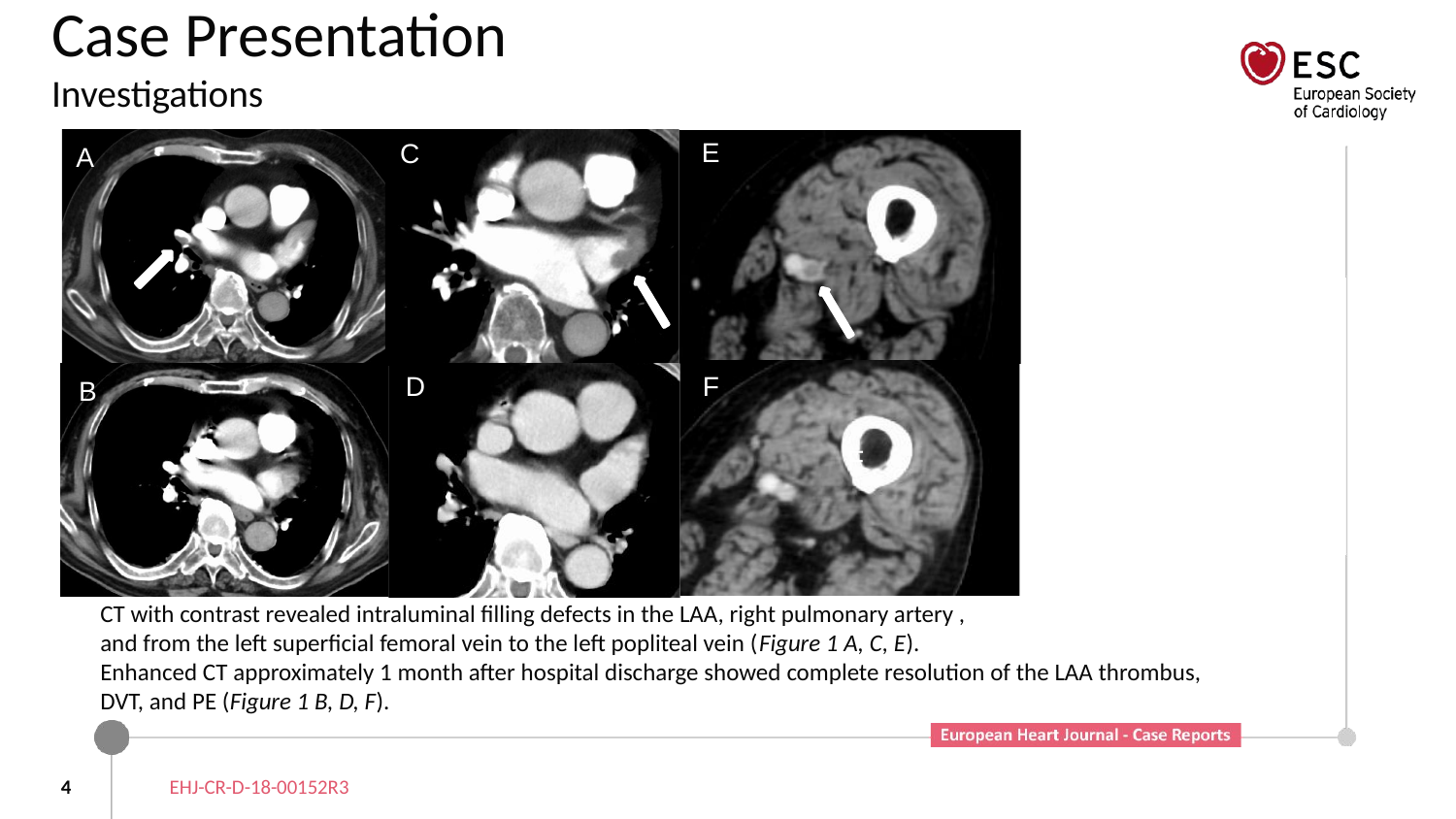

# Case Presentation Investigations
E
C
A
D
F
B
F
CT with contrast revealed intraluminal filling defects in the LAA, right pulmonary artery ,
and from the left superficial femoral vein to the left popliteal vein (Figure 1 A, C, E).
Enhanced CT approximately 1 month after hospital discharge showed complete resolution of the LAA thrombus,
DVT, and PE (Figure 1 B, D, F).
4
EHJ-CR-D-18-00152R3

## Slide 5
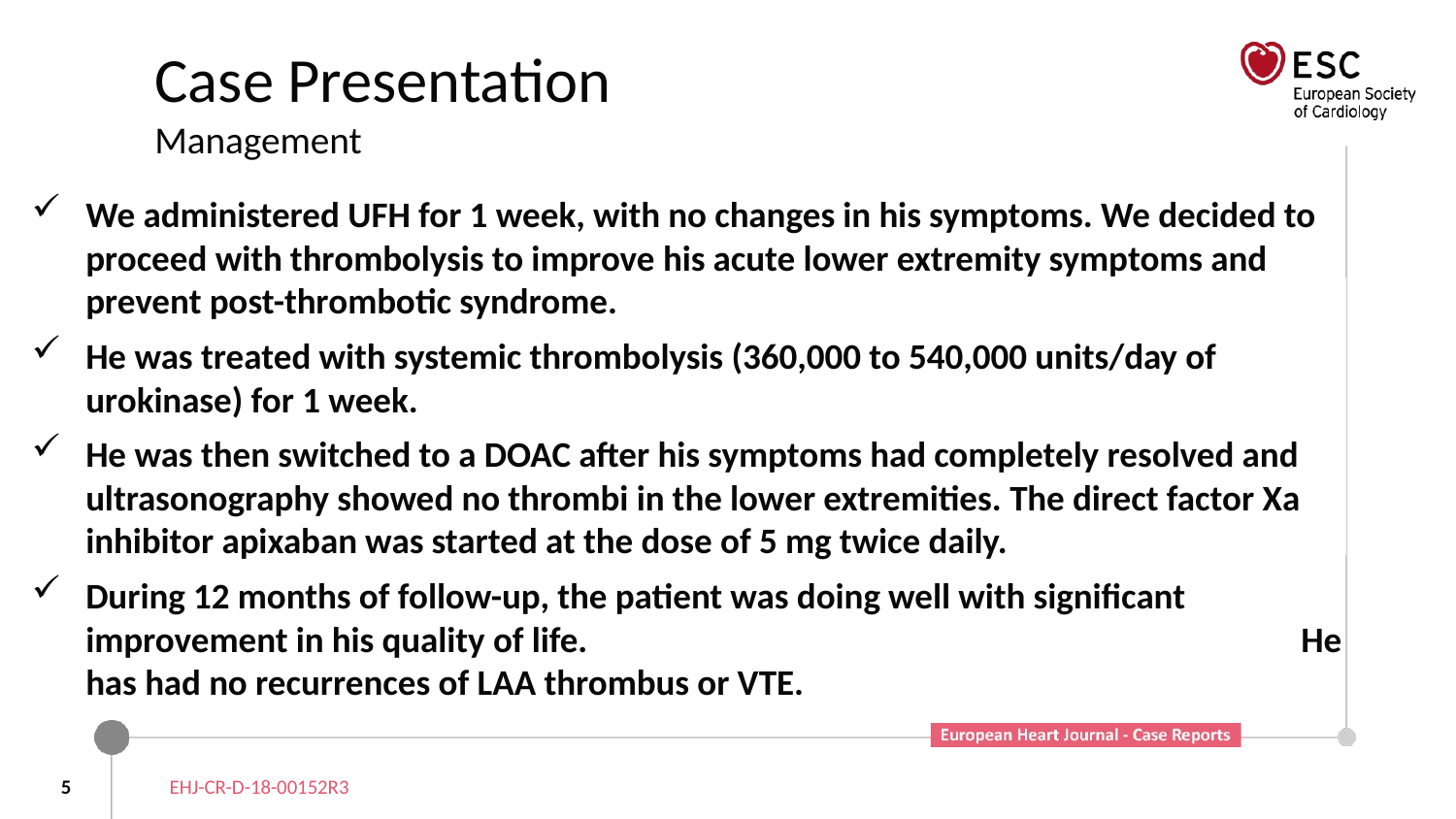

# Case Presentation Management
We administered UFH for 1 week, with no changes in his symptoms. We decided to proceed with thrombolysis to improve his acute lower extremity symptoms and prevent post-thrombotic syndrome.
He was treated with systemic thrombolysis (360,000 to 540,000 units/day of urokinase) for 1 week.
He was then switched to a DOAC after his symptoms had completely resolved and ultrasonography showed no thrombi in the lower extremities. The direct factor Xa inhibitor apixaban was started at the dose of 5 mg twice daily.
During 12 months of follow-up, the patient was doing well with significant improvement in his quality of life. He has had no recurrences of LAA thrombus or VTE.
5
EHJ-CR-D-18-00152R3

## Slide 6
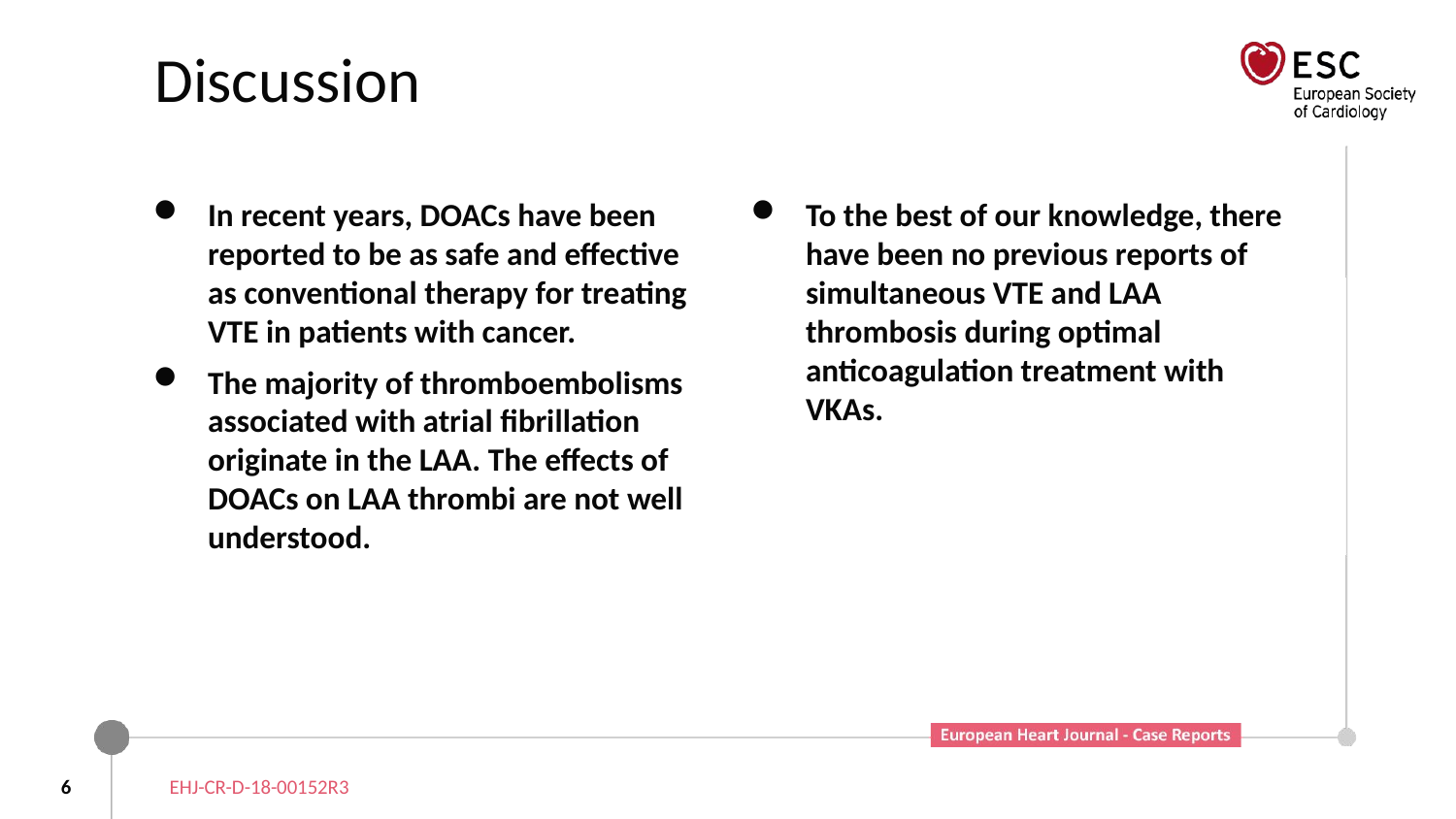

# Discussion
In recent years, DOACs have been reported to be as safe and effective as conventional therapy for treating VTE in patients with cancer.
The majority of thromboembolisms associated with atrial fibrillation originate in the LAA. The effects of DOACs on LAA thrombi are not well understood.
To the best of our knowledge, there have been no previous reports of simultaneous VTE and LAA thrombosis during optimal anticoagulation treatment with VKAs.
6
EHJ-CR-D-18-00152R3

## Slide 7
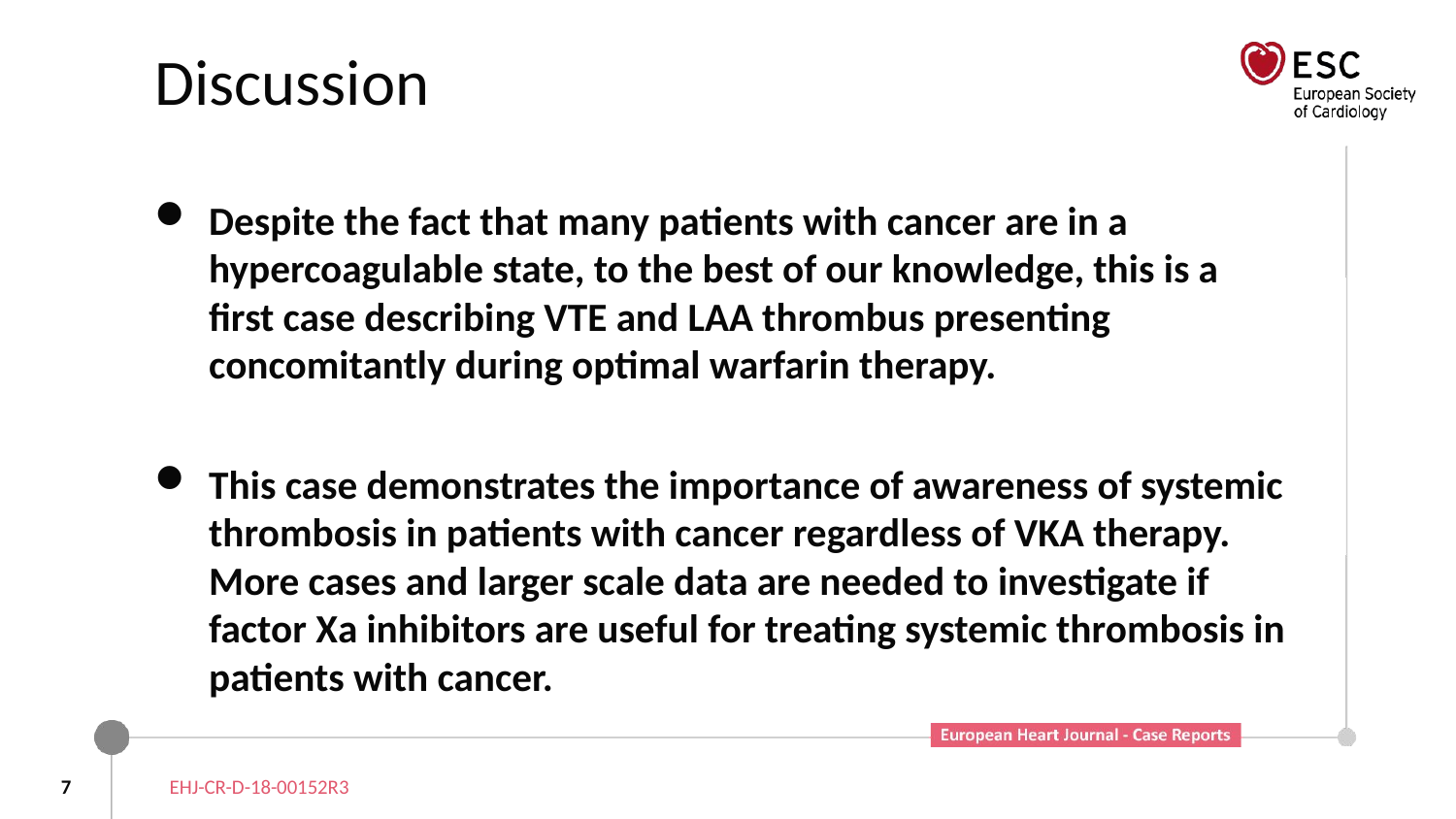

# Discussion
Despite the fact that many patients with cancer are in a hypercoagulable state, to the best of our knowledge, this is a first case describing VTE and LAA thrombus presenting concomitantly during optimal warfarin therapy.
This case demonstrates the importance of awareness of systemic thrombosis in patients with cancer regardless of VKA therapy. More cases and larger scale data are needed to investigate if factor Xa inhibitors are useful for treating systemic thrombosis in patients with cancer.
7
EHJ-CR-D-18-00152R3

## Slide 8
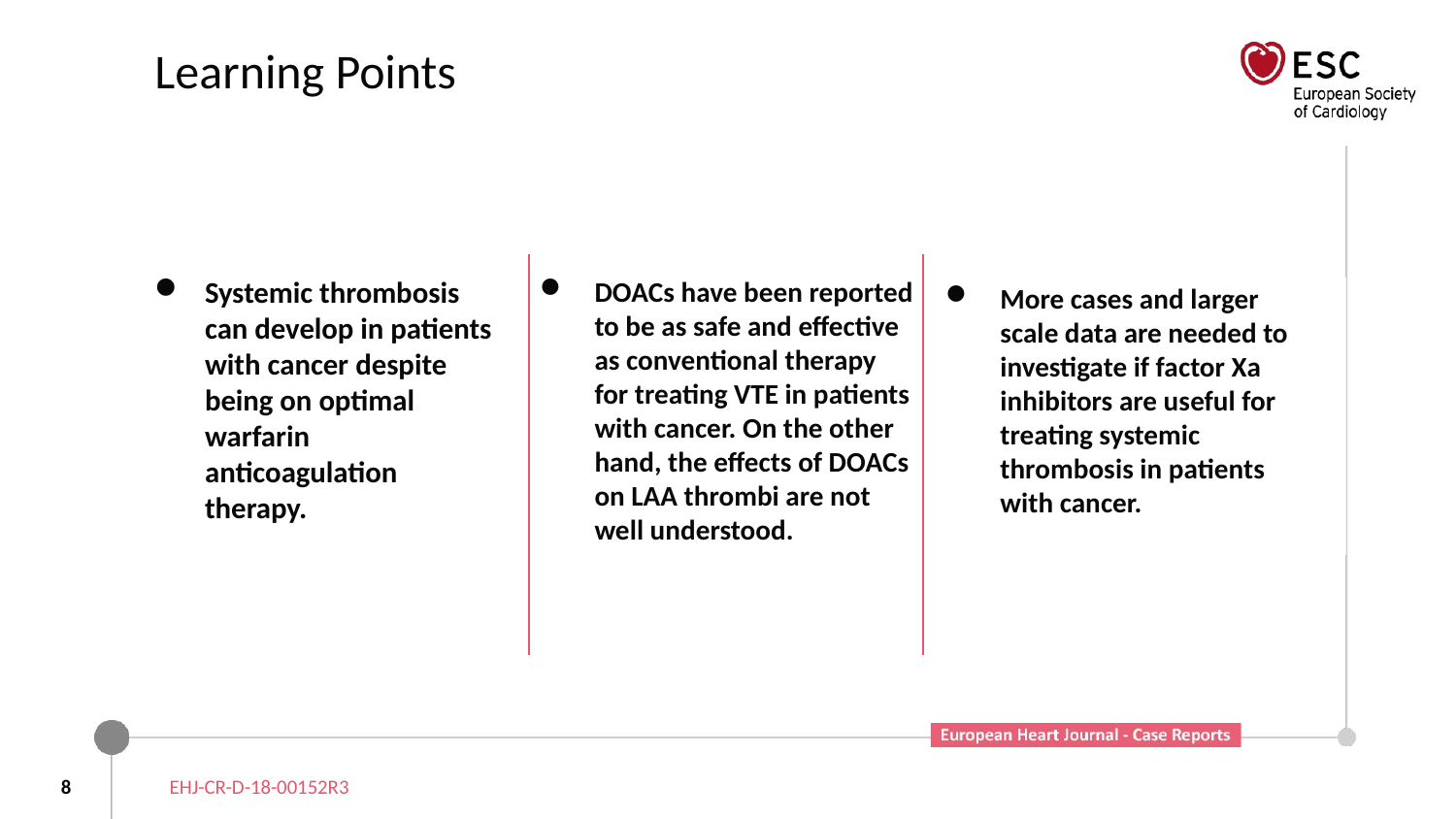

# Learning Points
Systemic thrombosis can develop in patients with cancer despite being on optimal warfarin anticoagulation therapy.
DOACs have been reported to be as safe and effective as conventional therapy for treating VTE in patients with cancer. On the other hand, the effects of DOACs on LAA thrombi are not well understood.
More cases and larger scale data are needed to investigate if factor Xa inhibitors are useful for treating systemic thrombosis in patients with cancer.
8
EHJ-CR-D-18-00152R3

## Slide 9
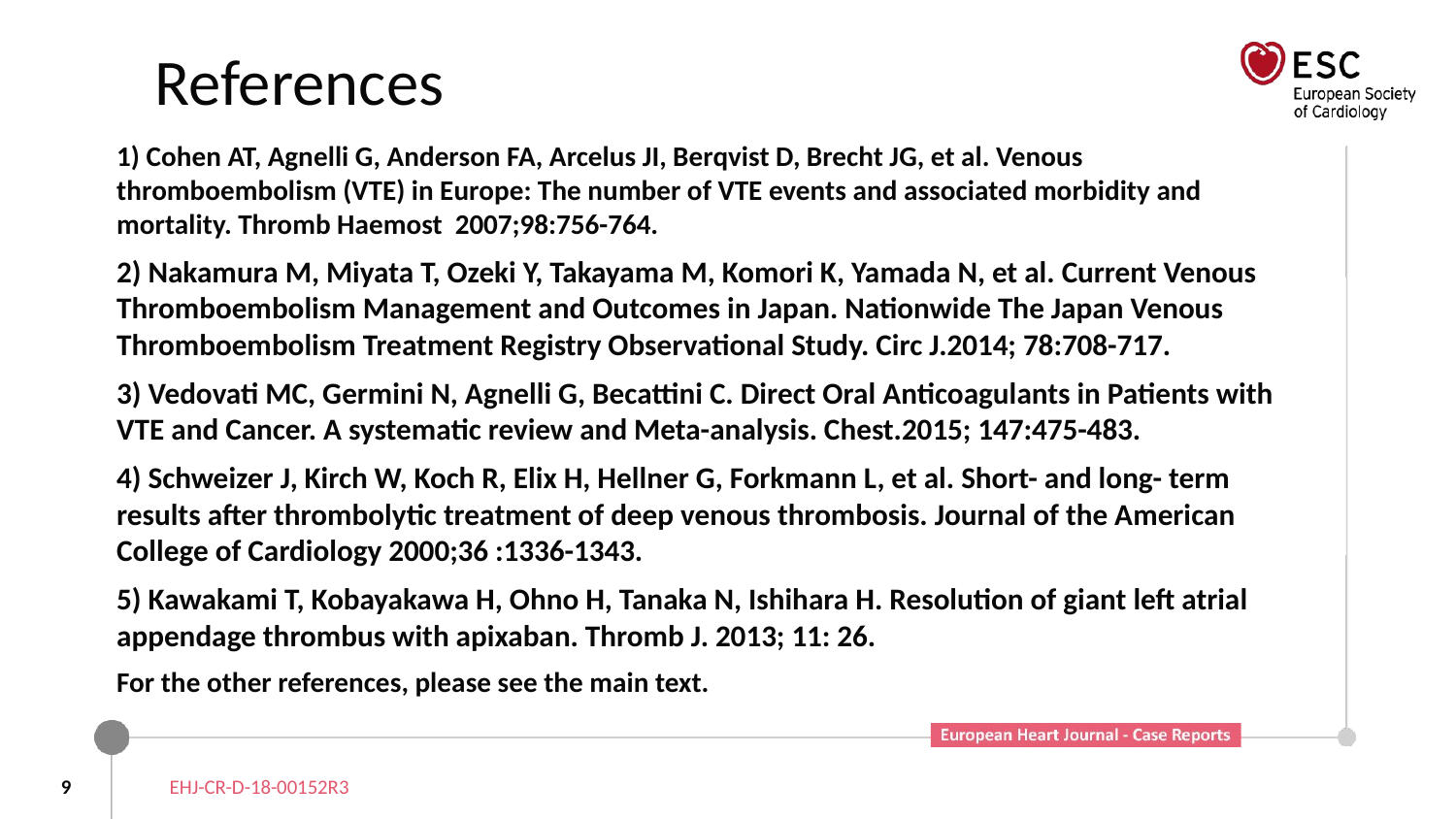

# References
1) Cohen AT, Agnelli G, Anderson FA, Arcelus JI, Berqvist D, Brecht JG, et al. Venous thromboembolism (VTE) in Europe: The number of VTE events and associated morbidity and mortality. Thromb Haemost 2007;98:756-764.
2) Nakamura M, Miyata T, Ozeki Y, Takayama M, Komori K, Yamada N, et al. Current Venous Thromboembolism Management and Outcomes in Japan. Nationwide The Japan Venous Thromboembolism Treatment Registry Observational Study. Circ J.2014; 78:708-717.
3) Vedovati MC, Germini N, Agnelli G, Becattini C. Direct Oral Anticoagulants in Patients with VTE and Cancer. A systematic review and Meta-analysis. Chest.2015; 147:475-483.
4) Schweizer J, Kirch W, Koch R, Elix H, Hellner G, Forkmann L, et al. Short- and long- term results after thrombolytic treatment of deep venous thrombosis. Journal of the American College of Cardiology 2000;36 :1336-1343.
5) Kawakami T, Kobayakawa H, Ohno H, Tanaka N, Ishihara H. Resolution of giant left atrial appendage thrombus with apixaban. Thromb J. 2013; 11: 26.
For the other references, please see the main text.
9
EHJ-CR-D-18-00152R3
